# Detailed Morphometric Analysis on Left Coronary Artery in the Population of North-East India

**DOI:** 10.7759/cureus.45023

**Published:** 2023-09-11

**Authors:** Chau Pingsaymang Manpoong, Bishwajeet Saikia, Mohan K Ram, Amitav Sarma, Amit Malviya

**Affiliations:** 1 Anatomy, North Eastern Indira Gandhi Regional Institute of Health and Medical Sciences (NEIGRIHMS), Shillong, IND; 2 Cardiology, North Eastern Indira Gandhi Regional Institute of Health And Medical Sciences (NEIGRIHMS), Shillong, IND

**Keywords:** coronary trifurcation, left coronary artery, left main trunk, left main artery, coronary artery disease, coronary angiography, anatomic variation, coronary circulation, coronary vessels

## Abstract

Introduction

The left ventricle, the cardiac chamber responsible for blood supply to the whole of systemic vasculature, receives most of its blood supply from the left coronary arteries (LCAs). Atherosclerosis of these vessels leading to myocardial infarction is a leading cause of death. Several invasive diagnostic or therapeutic coronary interventions are available for such patients. Just like any vascular procedure, a prior comprehensive knowledge of the dimensions of these vessels and their branching pattern is essential to perform these procedures uneventfully. No previous study in the population of North-Eastern India documents the population-specific reference for morphometric values of left coronary arteries and their anatomic variations. So, this study aims to fill up this lacuna.

Methods

This study was conducted in the Department of Anatomy in collaboration with the Catheterization Lab, Department of Cardiology, North Eastern Indira Gandhi Regional Institute of Health and Medical Sciences (NEIGRIHMS), Shillong. Coronary angiograms (CAG) of 100 subjects - 38 females and 62 males - were obtained from the Cardiac Catheterization Lab. Coronary angiograms were studied for the normal variant anatomy and morphometry of the LCAs - the left main coronary artery (LMCA), left anterior descending (LAD), and left circumflex (LCX).

Results

The mean length and luminal diameter of LMCA were found to be 9.13±3.23 mm and 4.38±0.58 mm, respectively. The mean length of LAD and LCX were 109.46±14.49 mm and 66.27±11.56 mm, respectively. Ramus intermedius was present in 32% of the subjects, whereas the remaining subjects had bifurcations of LMCA. We also found that 86% of patients had “wrap-around LAD”, while in 11% of our subjects, LAD failed to reach the apex. Diagonal branches originating from LAD were single, duplicated, and multiple in 14%, 62%, and 24% respectively. The marginal branches were found to be single, double, and multiple in 20%, 51%, and 29% respectively.

Conclusion

This study establishes a baseline reference on morphometry of the left coronary artery specific to the population of North-East India. This study may be of assistance to radiologists and cardiologists when performing procedures on the left coronary arteries in the population of North-Eastern India, with respect to the prevalence of anatomic variations.

## Introduction

Left coronary arteries provide 84% of blood flow to the left ventricle, and so, diseases related to a left coronary artery have an increased risk of death [1]. As such, coronary vascular procedures like balloon angioplasty, stent placement, and atherectomy are also very frequently performed. A basic knowledge of the coronary arteries' dimensions and their branching pattern is essential for the flawless performance of such techniques. The presence of a huge spectrum of anatomic variations in the morphometry of coronary arteries is expected to have an influence on the outcome of these procedures. Since these coronary arteries show significant morphometric variations, it is prudent to know the reference morphometric values for any population. In this cross-sectional study, we have attempted to establish baseline data for normal morphometry of the left coronary artery segments in vitro, using quantitative coronary angiography. This is the first such study to be documented in the population of North-East India.

## Materials and methods

With the approval of the Institutional Ethical Committee, normal coronary angiograms from 100 subjects above the age of 18 years were obtained from the Department of Cardiology at NEIGRIHMS, India, between September 2021 and June 2022. Our study included both sexes with a mean age of 53.4±11.82 years (range: 33 to 76 years). Exclusion criteria included the presence of CAD, history of congenital heart disease, bypass surgery, or hypersensitivity to iodine-based contrast agents. The coronary branches were identified and confirmed by a cardiologist. Once confirmed, various segments of the arteries were measured accordingly using the Allura Xper FD 10 (Philips Medical Systems, Best, The Netherlands).

**Figure 1:**
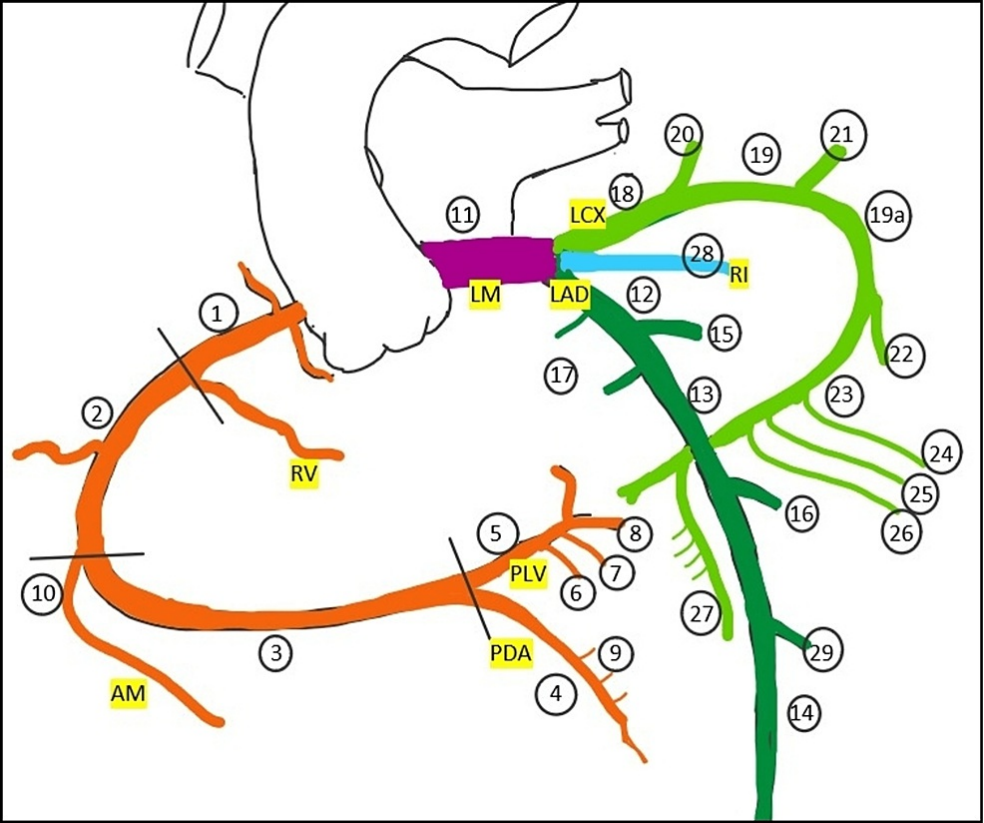
Schematic map of the coronary arterial tree. 1=Proximal Right Coronary Artery (RCA), 2=Mid RCA, 3=Distal RCA, 4=PDA from RCA, 5=Posterior Left Ventricular (PLV{ branch of the RCA, 6=1st PLV branch of RCA, 7=2nd PLV branch of RCA, 8=3rd PLV branch of RCA, 9=Inferior septal branches, 10=Acute Marginal branch of RCA, 11=Left main trunk of LCA, 12=Proximal LAD, 13=Mid LAD, 14=Distal LAD, 15=1st diagonal, 16=2nd diagonal, 17=Septal branches of the LAD, 18=Proximal LCX, 19=Mid LCX, 19a=Distal LCX, 20 =1st obtuse marginal, 21=2nd obtuse marginal, 22=3rd obtuse marginal, 23=LCX continuing as the left atrioventricular branch, 24=1st left PLV, 25=2nd left PLV, 26=3rd left PLV, 27=Left Posterior Descending Artery (in left-dominant circulation), 28=Ramus intermedius, 29=3rd diagonal LM=Left Main coronary trunk, LAD=Left Anterior Descending artery, LCX=Left Circumflex, RI=Ramus Intermedius, RV=arterial branch to Right Ventricle, AM=Acute Marginal branch, PDA=Posterior Descending Artery, PLV=Posterior Left Ventricular artery The figure was created by Dr. Chau Pingsaymang Manpoong.

The segments of the left coronary artery were described according to the classification proposed in the Bypass Angioplasty Revascularization Investigation (BARI) trial reported by the BARI Investigators** **(Figure 1) [2].

Coronary angiography and data analysis

Access for coronary angiography was established by percutaneous transfemoral route using the Seldinger technique. An arterial (5F/6F) sheath was inserted into the femoral artery and selective coronary catheterization was carried out with 4F/5F Judkins or Amplatz right and left coronary catheters. Selective hooking of the coronary ostium was performed, and 7-8 ml non-ionic Iodine contrast (Iohexol 350mg/ml) was administrated by hand injection.

Computed Tomography Angiography (CTA) images were then acquired for each coronary artery segment in two orthogonal views and the mean of the two values was taken for statistical analysis. The angiographic views with minimal foreshortening of involved coronary segments were selected. All the selected angiograms were reviewed by a cardiologist, both for the definition of normal vessels and subsequent quantitative analysis.

The coronary artery segment being studied was then delineated from adjacent intervening structures. The length and diameter of each artery were measured using a computer-assisted, inbuilt Quantitative Coronary Analysis software embedded in Allura Xper FD (Philips Medical Systems, Best, The Netherlands)**.**

Statistical analysis

The data was compiled and statistically analyzed using a Microsoft Excel sheet (Microsoft Corporation, Redmond, USA). Continuous data such as length, luminal diameter, and angle of bifurcation, were expressed as mean ± standard deviation.

## Results

All the subjects had two main coronary arteries, namely, the right and left coronary arteries.

Left main coronary artery

Length

The mean length of LMCA was found to be 9.13±3.23 mm (range 2-19.5 mm) (Table 1). The length of the left main trunk was between 5-10 mm in 67%, 10-15 mm in 20%, less than 5 mm in 7%, and more than 15 mm in 6% of the subjects. A total of 75% of the subjects had LMCA below 10.36 mm, whereas 25% had LMCA below 7.4 mm.

**Table 1:** Length and diameter of the major left coronary arteries (in mm) LMCA: Left Main Coronary Artery; LAD: Left Anterior Descending artery; LCX: Left Circumflex artery

Parameter	Mean ± Standard deviation	Range
Length of LMCA	9.13 ± 3.23	2 - 19.5
Length of LAD	109.46 ± 14.49	72.46 - 144.78
Length of LCX	66.27 ± 11.56	40.7 - 107.66
Luminal diameter of LMCA	4.38 ± 0.58	2.25 - 5.72
Luminal diameter of LAD	2.62 ± 0.5	1.69 - 4.06
Luminal diameter of LCX	2.41 ± 0.43	1.5 - 3.67

Luminal Diameter

The mean luminal diameter of the left main trunk was found to be 4.38±0.58 mm, with a minimum diameter of 2.25 mm and a maximum of 5.72 mm (Table 1). The luminal diameter was less than 4.02 mm in 25% of the subjects, whereas 25% of them had a luminal diameter of more than 4.76 mm.

Branching Pattern

**Figure 2:**
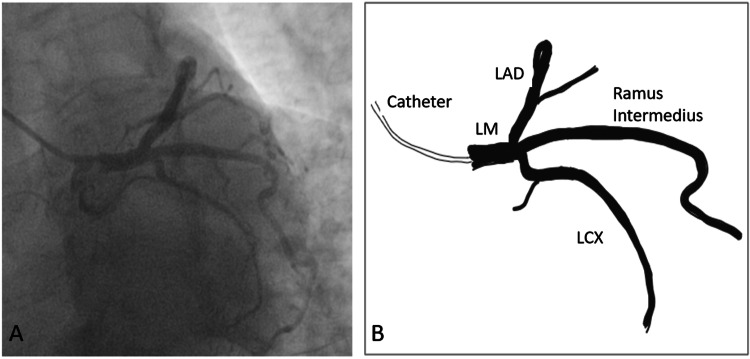
(A) Coronary angiography showing Ramus intermedius as seen in the Left Anterior Oblique view (Spider’s view). (B) Schematic illustration of the Ramus intermedius as seen in panel (A). LM: Left Main coronary trunk; LAD: Left Anterior Descending artery; LCX: Left Circumflex artery. Figure 2B created by Dr. Chau Pingsaymang Manpoong.

Bifurcations of the left main trunk were found in 68% of these subjects, whereas the remaining 32% showed a trifurcation pattern (with a Ramus Intermedius arising between LAD and LCX: Figure 2). No other termination pattern of the LMCA was seen.

LAD - LCX Bifurcation Angle

The mean angle between LAD and LCX was found to be 77.88±19.83°. In the study population, the angle between LAD and LCX ranged from 11.77° to 126°.

Left anterior descending artery

Length

The mean length of LAD was found to be 109.46±14.49 mm.

Luminal Diameter

The average luminal diameter of LAD was found to be 2.62±0.5 mm.

Diagonal Branches

**Figure 3:**
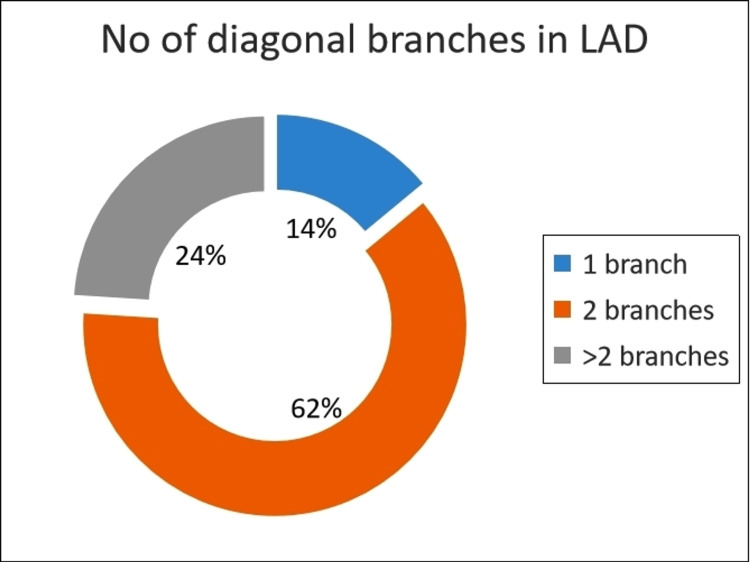
Pattern of the diagonal branches of the left anterior descending artery. LAD: Left Anterior Descending artery.

Double diagonal branches arising from LAD were seen in 62% of subjects, 14% had a single branch, and the remaining 24% had multiple (more than two) diagonal branches (Figure 3).

Termination

Wrap-around LAD - the LAD reaching the apex and wrapping around the apex to supply the apical segment of the diaphragmatic surface [3] - was seen in 86% of subjects. In 11% of our studied population, the LAD failed to reach the apex, while in 3% the LAD reached the apex but did not wrap around to supply the inferior segment of the apex.

Left circumflex artery

Length

The mean length of the LCX was found to be 66.27±11.56 mm.

Luminal Diameter

The average luminal diameter of the LCX was found to be 2.41±0.43 mm.

Marginal Branches

**Figure 4:**
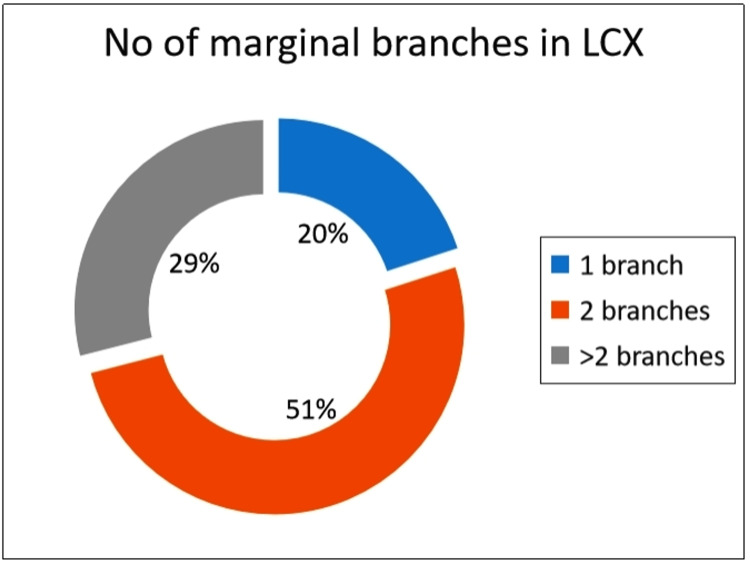
Pattern of the marginal branches of the left circumflex artery. LCX: Left Circumflex artery.

Two marginal branches originating from the LCX were seen in 51% of the subjects, 20% subjects had one branch, and the remaining 29% had more than two marginal branches (Figure 4).

## Discussion

Factors like genetics, age, gender, ethnicity, and body weight have been linked to coronary artery variations in many of the previous studies, and these variations have many crucial clinical consequences.

**Figure 5:**
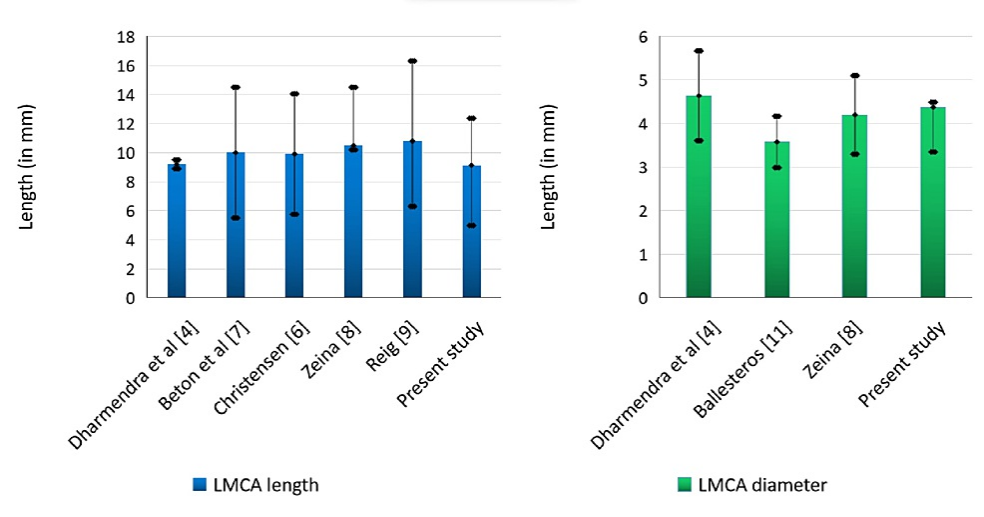
Comparison of the length and luminal diameter of LMCA from different studies. LMCA: Left Main Coronary Artery.

For instance, LMCA lengths less than 5 mm are considered to be short LMCA trunks and are associated with a high risk of injury when angiography is performed. Some cadaveric studies reported the mean length of LMCA to be 9.2±0.31 mm [4] and ranging from 5-20 mm [5]. Most CTA studies showed a relatively longer LMCA with a mean LMCA length of 9.9±4.15 mm [6], 10.0±4.5 mm [7], and 10.5±4 mm [8] (Figure 5).

The difference in the mean length of the main trunk of LCA could be due to the difference in methodology and study population. Otherwise, LMCA length seems to be quite stable. The least average mean length in LMCA of 9.13±3.23 mm (range: 2-19.5 mm) was observed in our study, which may be due to differences in the study population (Table 2). Even in our study, 7% of the population had an LMCA length of less than 5 mm and 5% of the population had a long LMCA trunk (LMCA length more than 15 mm).

**Table 2:** Comparison of LMCA lengths by different studies LMCA: Left Main Coronary Artery

Study	Methodology	Population	No of cases	Length of LMCA (mm)
Dharmendra (2013) [4]	cadaveric	India	93	9.2 ± 0.31
Alam (2017) [5]	cadaveric	Bangladesh	50	5-20 (Range)
Beton (2016) [7]	CT	Turkey	201	10.0 ± 4.5
Christensen (2010) [6]	CT	USA	105	9.9 ± 4.15
Zeina (2007) [8]	CT	Israel	70	10.5±4
Reig (2004) [9]	cadaveric	Spain	100	10.8 ± 5.52
Present Study	CT	India	100	9.13±3.23

Since the length of LMCA is inversely proportional to the risk of injury during angiography [10], it is crucial to know the baseline LMCA length in a population to plan and perform effective diagnostic and therapeutic interventions.

A coronary CTA study reported the cross-sectional diameters of LMCA at three different points, namely, at the level of the ostium, mid-portion, and distal portion as 5.3±1 mm, 4.2±0.9 mm, and 4.4±0.7 mm, respectively [8]. The minor increase in diameter of the distal portion of LMCA than the mid portion may be due to the bifurcation of the terminal part of LMCA. Two other cadaveric studies reported the mean LMCA diameter to be 3.58±0.59 mm [11] and 4.64±1.03 mm [4] (range 3 to 6.8 mm). The mean luminal diameter of LMCA measured in our study was 4.38±0.11 mm (Table 3).

**Table 3:** Comparison of LMCA luminal diameter by different studies LMCA: Left Main Coronary Artery

Study	Methodology	Population	Diameter of LMCA (mm)
Dharmendra (2013) [4]	cadaveric	India	4.64 ± 1.03
Ballesteros (2008) [11]	cadaveric	Colombia	3.58 ± 0.59
Zeina (2007) [8]	CT	Israel	4.20 ± 0.90
Present study	CT	India	4.38 ± 0.11

In every study listed in Table 4, the LMCA mostly terminated by dividing into two branches, and the next common termination pattern was a trifurcation. Out of these 11 studies, only two studies showed a pentafurcation pattern of LMCA termination. These studies also showed quadrification of LMCA, along with three more studies that only had a quadrification. Absent LMCA was also observed in a few studies.

**Table 4:** Comparison of LMCA termination pattern by different authors [5,6,10,12-18] LMCA: Left Main Coronary Artery

Study	Population	No of cases	Absent LMCA	Bifurcation	Trifurcation	Quadrification	Pentafurcation
Priya (2021) [10]	India	50	4	60	30	6	-
Fatima (2021) [12]	India	76	-	81.5	14.5	4	-
Kumar (2018) [13]	Oman	78	-	80.76	10.25	7.69	1.28
Lakshmiprabha (2018) [14]	India	55	-	54.54	41.82	1.82	1.82
Alam (2017) [5]	Bangladesh	50	-	74	26	-	-
Hosapatna (2013) [15]	India	30	-	93.3	6.7	-	-
Kulkarni (2012) [16]	India	107	2	86.46	11.54	-	-
Koşar (2009) [17]	Turkey	700	-	69	31	-	-
Christenen, 2009 [6]	USA	105	-	81	19	-	-
Agnihotri (2013) [18]	India	100	-	66	30	4	-
Present study	India	100	-	68	32	-	-

**Figure 6:**
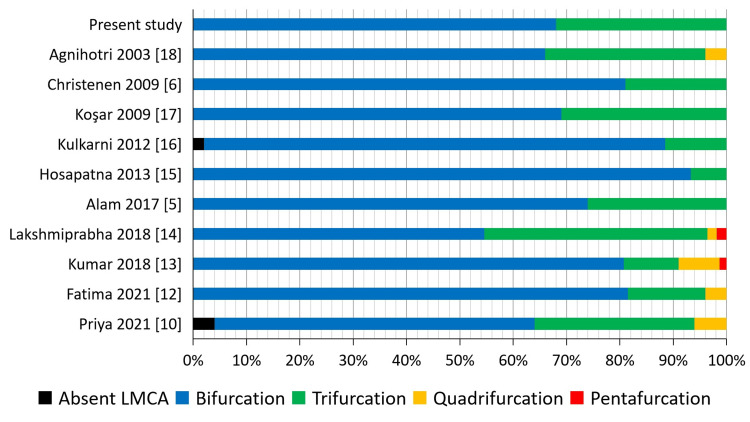
Comparing the termination pattern of LMCA by different studies. LMCA: Left Main Coronary Artery.

In the present study, a bifurcation pattern was found in 68% of subjects and trifurcation in 32% of subjects; no other pattern was seen. The LMCA termination pattern of our study was very similar to the study done by Koşar et al [17]. Also, the study methodology of both these studies was angiographic studies, one being a standard angiogram and the other a CT angiogram (Figure 6).

Patients with a trifurcation pattern of LMCA termination are more prone to stent thrombosis after certain coronary procedures than patients with a bifurcation pattern. So, it is clinically mandatory to know the prevalence of each variant of LMCA termination pattern. There is a relative paucity of literature on the clinical implications of quadrification and pentafurcation patterns of LMCA termination due to their reduced incidence [19].

**Figure 7:**
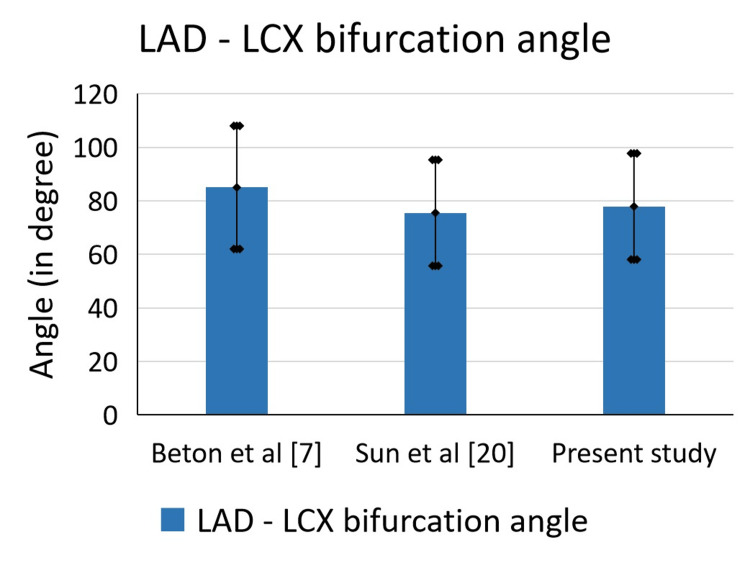
Mean and standard deviation of LAD - LCX bifurcation angle from different studies LAD: Left Anterior Descending artery; LCX: Left Circumflex artery.

Besides the LMCA termination pattern, LAD - LCX bifurcation angle has its own clinical implications. There’s a direct correlation between the bifurcation angle and the formation of plaques leading to atherosclerosis [20]. Studies combining normal as well as diseased subjects found LAD - LCX bifurcation angle to be 85°±23° [7] and 89.1°±13.1° [20]. While the same study observed LAD - LCX bifurcation angle in normal and diseased subjects to be 75.5°±19.8° and 94°±19.7°, respectively (range: 55.3° - 134.5°) [20]. The mean LAD - LCX bifurcation angle in our study was found to be 77.88° (ranging from 11.77° to 126°), which is similar to the observations seen earlier in normal subjects (Figure 7).

**Figure 8:**
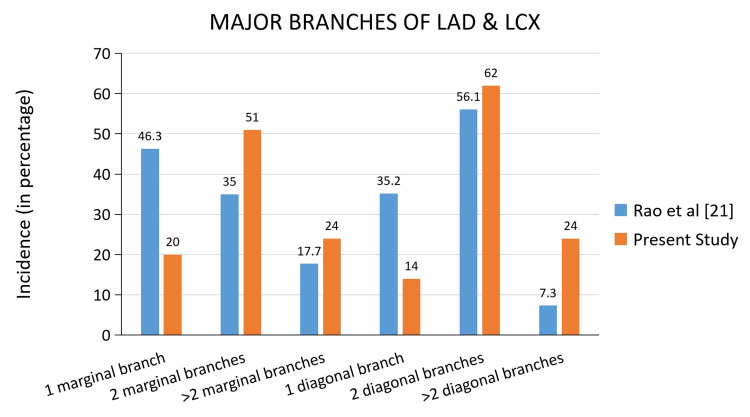
Comparison of the number of marginal and diagonal branches LAD: Left Anterior Descending artery; LCX: Left Circumflex artery.

The given bar chart (Figure 8) compares the number of marginal and diagonal branches of our study with a study by Rao et al. [21]. While some studies have reported the presence of more than one diagonal and marginal branch, other studies [22] reported the absence of diagonal or marginal branches.

**Figure 9:**
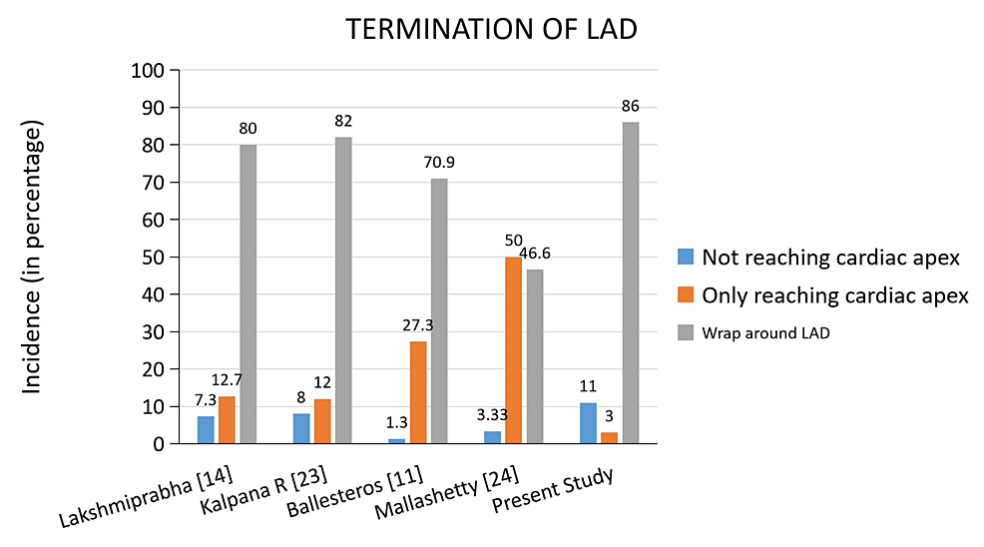
Comparison of the LAD termination pattern in different studies LAD: Left Anterior Descending artery.

The termination pattern of LAD has clinical relevance when performing reperfusion interventional procedures [10]. When comparing the LAD termination pattern of our study with a few other studies, “wrap-around LAD” seems to be the most common termination pattern. While LAD reaching the apex and not wrapping around was the next common variation in other studies (Figure 9) [11,14,23,24]. Termination of LAD even without reaching the apex was the second most common termination pattern of LAD (11%) in our study.

The third coronary artery was observed in other cadaveric studies, the presence of which ranged from 1% to 15% [12,25,26]. The present study did not find any third coronary artery in any of the subjects.

Limitations

The low sample size prevented us from performing morphometric analysis based on gender-based differences.

## Conclusions

The present study provides detailed baseline reference values concerning the morphometry of left coronary vasculature for the North-East Indian population and documents a wide range of variations. The mean length of LMCA can affect the outcome of coronary procedures and was found to be relatively smaller in the North-East Indian population. The luminal diameter of LMCA, LAD - LCX bifurcation angle, and the branching patterns of left coronary arteries seem normal and comparable with other studies. Patterns of LAD termination can affect the reperfusion procedures, and the present study found LAD termination before reaching the apex in 11% of the subjects. The data presented in this study is expected to provide better insight to cardiologists and radiologists when performing procedures on the left coronary arteries. 

Further studies incorporating the right coronary arteries and a bigger sample size should provide better evidence for normal and variant morphology in coronary vasculature in the North-East Indian population.

## References

[1] Collet C, Capodanno D, Onuma Y (2018). Left main coronary artery disease: pathophysiology, diagnosis, and treatment. Nat Rev Cardiol.

[2] The BARI Investigators (1991). Protocol for the bypass angioplasty revascularization investigation. Circulation.

[3] Villa AD, Sammut E, Nair A, Rajani R, Bonamini R, Chiribiri A (2016). Coronary artery anomalies overview: the normal and the abnormal. World J Radiol.

[4] Dharmendra P AT, Madan S, Londhe P (2013). Clinically significant variations of the left coronary artery in human cadaveric hearts. Int J Cur Res Rev.

[5] Alam SR (2017). Variations in the left coronary artery. CMOS Hosp Med Coll J.

[6] Christensen KN, Harris SR, Froemming AT, Brinjikji W, Araoz P, Asirvatham SJ, Lachman N (2010). Anatomic assessment of the bifurcation of the left main coronary artery using multidetector computed tomography. Surg Radiol Anat.

[7] Beton O, Kaplanoglu H, Hekimoglu B, Yilmaz MB (2017). Anatomic assessment of the left main bifurcation and dynamic bifurcation angles using computed tomography angiography. Folia Morphol (Warsz).

[8] Zeina AR, Rosenschein U, Barmeir E (2007). Dimensions and anatomic variations of left main coronary artery in normal population: multidetector computed tomography assessment. Coron Artery Dis.

[9] Reig J, Petit M (2004). Main trunk of the left coronary artery: anatomic study of the parameters of clinical interest. Clin Anat.

[10] Priya SJ, Sangeetha A, Krishna MS (2021). A cadaveric study on coronary artery variations and its clinical significance. Int J Sci Res.

[11] Ballesteros LE, Ramirez LM (2008). Morphological expression of the left coronary artery: a direct anatomical study. Folia Morphol (Warsz).

[12] Fatima N, Vivekanand Vivekanand, Kumari A, Sinha BK (2021). Study of branching, dominance pattern of coronary arteries in human cadaver. Eur J Mol Clin Med.

[13] Kumar A, Ajmani ML, Klinkhachorn PS (2018). Morphological variation and dimensions of left coronary artery: a cadaveric study. MOJ Anat Physiol.

[14] Lakshmiprabha S, Afroze KH, Ramesh P, Asha KR, Shivaleela C, Anupama D (2018). Variations in the anatomical and branching pattern of the left coronary artery: a cadaveric study. Int J Res Med Sci.

[15] Hosapatna M, D'Souza AS, Prasanna LC, Bhojaraja VS, Sumalatha S (2013). Anatomical variations in the left coronary artery and its branches. Singapore Med J.

[16] Kulkarni JP, Mehta L (2012). Main left coronary artery system - angiographic anatomy. J Dent Med Sci.

[17] Koşar P, Ergun E, Oztürk C, Koşar U (2009). Anatomic variations and anomalies of the coronary arteries: 64-slice CT angiographic appearance. Diagn Interv Radiol.

[18] Agnihotri G, Kaur M, Kalyan GS (2013). Branching patterns of left coronary artery among North Indians. Anat J Af.

[19] Shammas NW, Dippel EJ, Avila A (2007). Long-term outcomes in treating left main trifurcation coronary artery disease with the Paclitaxel-eluting stent. J Invasive Cardiol.

[20] Sun Z, Cao Y (2011). Multislice CT angiography assessment of left coronary artery: correlation between bifurcation angle and dimensions and development of coronary artery disease. Eur J Radiol.

[21] Rao A, Pimpalwar Y, Yadu N, Yadav RK (2017). A study of coronary artery variants and anomalies observed at a tertiary care armed forces hospital using 64-slice MDCT. Indian Heart J.

[22] Bhele AV, Ughade HM, Shaikh S, Joge US (2017). A study of course, branches and variations of the coronary arteries in the human cadaveric heart. Int J Contemp Med Res.

[23] Kalpana RA (2003). A study on principal branches of coronary arteries in humans. J Anat Soc Ind.

[24] Mallashetty N, Itagi V (2017). The study of branching pattern and variations in the left coronary artery in human heart with a unique case of crossing of coronary arteries- a cadaveric study. Ind J Clin Anatomy Physiol.

[25] Meshram SW LA, Rukhmode VP, Gajbe UL (2017). Multiple coronary ostia and their clinical and surgical significance. IOSR J Dent Med Sci.

[26] Chougule P, Silotry N, Chavan L (2014). Variation in branching pattern of coronary arteries. Int J Sci Res.

